# MVPA Is Associated with Lower Weight Gain in 8–10 Year Old Children: A Prospective Study with 1 Year Follow-Up

**DOI:** 10.1371/journal.pone.0018576

**Published:** 2011-04-28

**Authors:** Abigail Fisher, Claire Hill, Laura Webber, Lisa Purslow, Jane Wardle

**Affiliations:** Cancer Research UK Health Behaviour Research Centre, Department of Epidemiology and Public Health, University College London, London, United Kingdom; Pennington Biomedical Research Center, United States of America

## Abstract

**Background:**

Studies relating physical activity (PA) to weight gain in children have produced mixed results, although there is some evidence for stronger associations with more intense physical activities. The present study tested the hypothesis that weight gain over one year in 8–10 year olds would be more strongly predicted by moderate and vigorous physical activity (MVPA) than total physical activity (total PA) or sedentary behaviour.

**Methodology:**

Participants were 280 children taking part in the Physical Exercise and Appetite in Children Study (PEACHES). Weight status was assessed using body mass index (BMI), fat mass index (FMI), and waist circumference (WC) in school Year 4 (baseline; age 8.7 yrs) and Year 5 (follow-up; age 9.7 yrs). Physical activity was measured at baseline using the Actigraph GT1M accelerometer to assess total PA (mean accelerometers counts per minute), MVPA; ≥4000 counts per minute) and sedentary time (<100 counts per minute).

**Principal Findings:**

After adjustment for baseline BMI, SES, sex and ethnicity, MVPA was significantly associated with follow–up BMI (adjusted β = −0.07; p = 0.002). This association was independent of total PA or sedentary time. Similar results were observed for FMI; again MVPA was significantly associated with follow up FMI (β = −0.16; p = 0.001) independent of total PA or sedentary time. The pattern was similar for WC (β = −0.07), but the association between MVPA and WC did not reach significance at p = 0.06.

**Conclusion:**

The results of this study strongly support promotion of MVPA in children.

## Introduction

Prevention of childhood overweight and obesity is a public health priority [1]. Weight has a strong genetic influence [2], but the rapid rise in obesity prevalence over the past three decades points to an important role for the environment [3]. As a component of the energy balance equation, low physical activity (PA) is a plausible determinant of weight gain, but results in adults have been equivocal [4], [5]. However, studies that have focused specifically on high intensity physical activity have found more consistent associations with obesity risk [4], [5]. Childhood is important because habits related to energy balance may be established at this stage. There are relatively few studies examining associations between objectively-measured physical activity and weight status in childhood [5]–[7], but as in adults, some cross-sectional studies have found stronger associations with more vigorous activities [8]–[11].

Longitudinal designs provide a better basis for evaluating causal associations than cross-sectional studies. In the Avon Longitudinal Study of Parents and Children (ALSPAC), data from a very large sample of adolescents (n = 4150) showed that physical activity measured by accelerometer was associated with change in fat mass two years later; with time spent in moderate and vigorous physical activity (MVPA; defined as >3600 counts accelerometer counts per minute) being particularly important [12]. One of the few studies in younger children (age 4–6 yrs) found no association between combined moderate and vigorous activity (MVPA; defined as >2000 accelerometer counts per minute) and either body mass index or skinfold thickness [13]. However, in a sample of 4–6 year olds in the US, higher VPA was associated with lower adiposity three years later [14].

Rates of childhood obesity are increasing fastest in UK 8–10 year-olds [15] making this an important target age-group. In a previous study, we reported cross-sectional associations between objectively measured physical activity and weight status in a socioeconomically and ethnically diverse sample of 8–9 year olds, although the associations were only significant in boys [16]. This paper reports associations between physical activity (total daily PA, time spent in MVPA) and sedentary time at baseline and three measures of adiposity (BMI, FMI and WC) one year later, and specifically tests the prediction that MVPA would show the strongest association.

## Methods

### Participants

**Figure 1:**
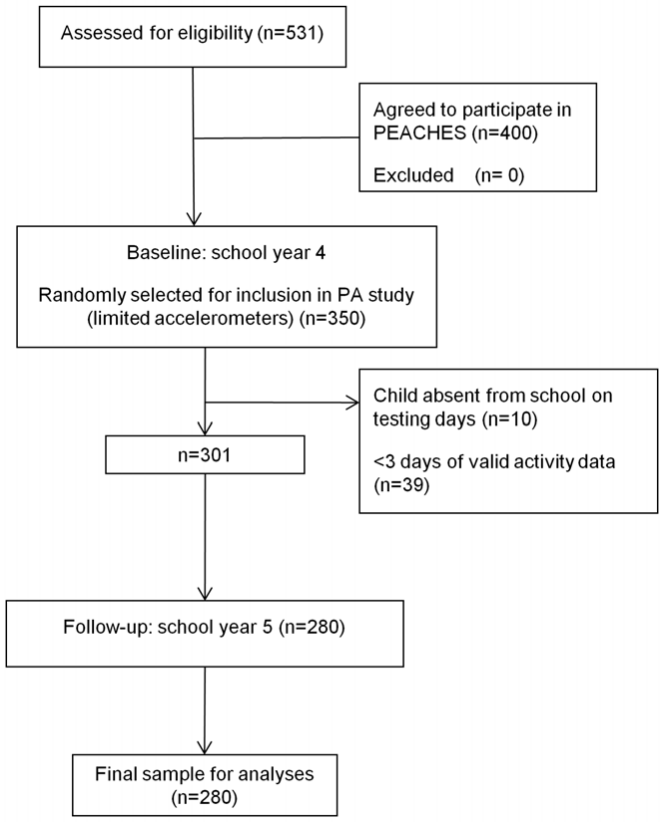
Flow of participants through the PEACHES physical activity study. There were no significant differences in sociodemographic data between those who provided full valid data at both time points and those who did not (p's all >0.05).

Participants were recruited as part of the Physical Exercise and Appetite in Children Study (PEACHES); a longitudinal study examining associations between appetite, physical activity and weight. Parents of all children in Years 3 and 4 in five schools in London, UK (n = 531) were sent information sheets and consent forms, and 400 (75%) consented to their child taking part; of whom 350 were present at school in Year 4 for baseline measurements. Flow of participants through the PEACHES physical activity study is presented in **Figure 1**. There were no differences in anthropometric or demographic measures between children who provided complete valid data and those who did not (all p's>0.05). The study was granted ethical approval by the University College London Committee on the Ethics of Non-NHS Human Research.

### Demographic data

Date of birth, sex and ethnicity data for participating children were provided by schools. Reported ethnicities were white (46%) black British (22%), Asian (12%) mixed race (8%), and ‘other’ (Iranian, South American, Afghani;12%) ; which were recoded as ‘white’ or ‘non-white’ for analysis because of the relatively small numbers in each ethnic sub-group. Socioeconomic status (SES) was indexed with area-level socioeconomic deprivation using the Townsend index [17] which is derived by matching residential postcodes to census data. A value of zero represents the national average deprivation level and a positive value indicates higher than average deprivation.

### Adiposity

Weight (measured to the nearest tenth of a kilogram) and fat mass (from leg-to-leg bioelectrical impedance) were assessed with the child wearing light clothing using the Tanita TBF-300MA Body Composition Analyser (Tanita Corporation, Tokyo, Japan). Height was measured without shoes to the nearest millimetre using a Leicester height measure (SECA, Birmingham, UK). Height and weight were used to calculate BMI (weight in kg/height in m^2^). Issues have been raised with presenting fat as a proportion of body weight in children, since percent fat is dependent on both fat and lean tissue, and therefore variation over time could arise from changes in either fat-free mass or fat mass. Adjusting fat mass for height (FMI) has been suggested as a solution, therefore FMI (calculated as fat mass/height in m^2^) was used in this study [18], [19]. Waist circumference (WC) was measured to the nearest millimetre according to standard instructions. To characterise the sample, BMI and WC were converted into age- and sex-appropriate standard deviation scores (SDS) using the lmsgrowth macro from (http://homepage.mac.com/tjcole) based on British 1990 reference data [20]. FMI was not converted as (to our knowledge) reference data are not available. For longitudinal analyses, we used raw rather than age- or gender-adjusted measures of adiposity as recommended [21]. In a subsample of 57 children, the anthropometric measures showed excellent inter-rater reliability (BMI r>0.99, FM r>0.99; WC r = 0.96).

### Physical activity

Physical activity was measured with the Actigraph GT1M accelerometer (www.theactigraph.com). This is the most widely used accelerometer in paediatric research [22] and has been validated against doubly-labelled water [23]. Physical activity variables used in this study were total physical activity (total PA; mean accelerometer counts per minute) and combined moderate and vigorous activity (MVPA: using a cut point of >4000 counts per minute). It has recently been proposed that cut points to define MVPA should not be set lower than 3000 counts per minute in 6–12 year old children [24]. Facilities were not available to carry out a calibration study in PEACHES. However, a previous calibration study in adolescents from the ALSPAC cohort suggest that >3581 counts per minute was approximately equivalent to moderate activity (4 times resting metabolic rate; METS) [25]. In 8–11 year olds, treadmill walking at 4.8 km/h produced an accelerometer value of 3425 counts per minute [26]. Since these cut points were developed using the older Actigraph 7164, which registers activity about 9% higher in children than the newer GT1M model used in this study [27], we decided that a cut-off of >4000 counts per minute would adequately capture MVPA in this group. Children wore the accelerometer around the waist on the right hip for five consecutive days including two weekend days; removing it only for bathing, sleeping and swimming. Activity data were recorded in one minute epochs and processed using the MAHUffe programme (www.mrcepid.cam.ac.uk/Research/PA/Downloads). Children were considered to have valid data provided the monitor was worn for at least three days including one weekend day, and a minimum of 600 minutes of valid data per day were recorded [25], [28]. Sedentary time was classified as activity counts per minute <100. In line with other studies, we distinguished sedentary behaviour from time when the monitor was not worn by excluding periods of >10 minutes with continuous zeros [25].

### Statistical analyses

Previous analysis of Year 4 (baseline) data had found higher PA in boys than girls and significant cross-sectional associations between sedentary behaviour, total PA and combined MVPA, and weight status in boys but not girls [16]. We therefore tested activity-by-gender interactions in relation to one-year change in adiposity, but they were non-significant (p's≥0.06). We also repeated the analyses stratifying by gender, and the results were the same in each gender group. Therefore data from the whole group were combined for these analyses and gender, SES and ethnicity were included as covariates.

Longitudinal associations between the anthropometric measures (BMI, FMI, WC) and physical activity were examined using hierarchical multiple regression. Step 1 tested each adiposity measure (BMI, FMI and WC) individually in a model including SES, ethnicity and gender and the baseline value for BMI, FMI and WC respectively. In Step 2, total PA, MVPA and sedentary time were added to the model to test for independent associations with the adiposity measures. Correlations between total PA, MVPA and sedentary time were low to moderate (r = 0.21–0.63) suggesting these behaviours were to some extent independent. For illustration purposes one year change in BMI, FMI and WC by tertiles of MVPA (controlling for age, gender, SES, ethnicity, total PA and sedentary behaviour) assessed using analysis of variance; ANOVA) were presented graphically. Correction for clustering effects was not needed as the intraclass correlations between school and MVPA were below the conventional value of 0.05 (ICC = 0.04). Data were analysed using SPSS (Version 15; SPSS Inc., Chicago, IL, USA). Alpha was set at p<0.05.

## Results

Characteristics of participants are presented in **Table 1**. As expected, BMI, FMI and WC were all significantly greater at follow-up than baseline (all p's<0.05), and the rise in BMI and waist SD scores indicated increasing adiposity compared with the standardisation data. Only 1% of children in the sample met even the minimum guidelines of at least 60 minutes of MVPA [29], and the total amount of time spent in MVPA was very low; averaging only 12 minutes per day.

**Table 1:** Participant characteristics (n = 280).

	Year 4 (baseline)	Year 5 (follow-up)
Age (years)	8.75 (0.37)	9.72 (0.37)**
Body mass index (BMI)	16.94 (2.90)	17.52 (3.20)**
BMI s.d.†	0.11 (1.53)	0.15 (1.32)
Fat mass index (FMI)*	4.42 (3.05)	6.07 (3.05)**
Waist circumference (cm)	60.55 (6.48)	63.34 (7.83)**
Waist s.d.†	0.87 (1.04)	1.00 (1.07)**
Townsend Index (SES)	1.64 (2.13)	1.64 (2.13)
Sex (%)		
Male	51	51
Female	49	49
Ethnicity (%)		
White	46	46
Black British	22	22
Asian	12	12
Mixed race	8	8
Other	12	12
Physical activity		
Total PA (mean counts/min)	604 (156)	-
Daily minutes of MVPA (>4000/min)	12 (10)	-
Daily minutes spent sedentary	330 (119)	-

Data are from 280 children who were present in school years 4 and 5.

Values are means and standard deviations unless otherwise stated.

† Age- and gender-adjusted standard deviation scores relative to the 1990 UK reference data.

**significantly higher than baseline at p<0.001.

*FMI n = 278.

Ethnicities coded as other included Iranian, South American, Afghani).

Results of hierarchical regressions are presented in **Tables 2** **,** **3** **,** **4**. Baseline BMI, SES, sex and ethnicity (Step 1) explained 92% of the variance in follow-up BMI. The addition of the physical activity and sedentary time variables (Step 2) was significant (p = 0.02) and explained an additional 1% of the variance. MVPA was the only significant activity variable in the model (p = 0.002). MVPA was negatively associated with BMI, suggesting that higher levels of MVPA at baseline were associated with lower follow-up BMI one year later. Similar results were observed for FMI; Step 1 explained 72% of the variance and the addition of Step 2 explained an additional 1% (p = 0.01). Again only MVPA was significantly (negatively) associated with follow-up FMI in Step 2 (p = 0.001). The variables in Step 1 also explained a large proportion of the variance in follow-up WC (83%) and Step 2 explained an additional 1%, although, the addition did not reach significance (p = 0.06).

**Table 2:** Associations between physical activity and BMI one year later (n = 280).

	b	SE	β	95% CI B
Step 1				
Baseline BMI	1.06**	0.02	0.96**	1.03, 1.10
SES	−0.02	0.02	−0.12	−0.05, 0.01
Sex	−0.33*	0.12	−0.05*	−0.53, −0.12
Ethnicity	−0.10	0.12	−0.02	−0.32, 0.11
Step 2				
MVPA	−0.02*	0.01	−0.07*	−0.04, −0.01
Total physical activity	0.01	0.01	0.04	−0.01, 0.02
Sedentary time	0.01	0.00	0.03	0.00, 0.01

Data are from 280 8–10 year-old children. BMI = body mass index.

MVPA = moderate and vigorous physical activity (>4000 accelerometer counts per minute). Sedentary time (<100 accelerometer counts per minute).

Hierarchical linear regression: b = unstandardised Beta value, β = standardised beta.

Adjusted R^2^ for Step 1 = 0.92, p<0.001; Adjusted R^2^ for Step 2 = 0.93, Δ R^2^ p = 0.02.

**Table 3:** Associations between physical activity and FMI one year later (n = 279).

	b	SE	β	95% CI B
Step 1				
Baseline FMI	0.63**	0.26	0.85**	0.58, 0.68
SES	−0.01	0.22	−0.01	−0.05, 0.04
Sex	0.03	0.15	0.01	−0.27, 0.33
Ethnicity	−0.03	0.17	−0.01	−0.36, 0.29
Step 2				
MVPA	−0.04*	0.01	−0.16*	−0.06, 0.01
Total physical activity	−0.01	0.01	0.09	0.00, 0.03
Sedentary time	0.00	0.01	−0.02	−0.02, 0.01

Data are from 280 8–10 year-old children. FMI = fat mass index.

MVPA = moderate and vigorous physical activity (>4000 accelerometer counts per minute).

Sedentary time (<100 accelerometer counts per minute).

Hierarchical linear regression: b = unstandardised Beta value, β = standardised beta.

Adjusted R^2^ for Step 1 = 0.72, p<0.001; Adjusted R^2^ for Step 2 = 0.73, Δ R^2 p = ^ p = 0.01.

**Table 4:** Associations between physical activity and WC one year later (n = 280).

	b	SE	β	95% CI B
Step 1				
Baseline WC	1.06**	0.03	0.90	0.99, 1.11
SES	−0.05	0.06	−0.03	−0.17, 0.06
Sex	−0.31	0.40	−0.02	−1.10, 0.48
Ethnicity	−0.54	0.43	−0.04	−1.38, 0.31
Step 2				
MVPA	−0.05	0.03	−0.07	−0.12, 0.01
Total physical activity	0.01	0.02	0.03	−0.02, 0.01
Sedentary time	−0.02	0.02	−0.03	−0.01, 0.02

Data are from 280 8–10 year-old children participating in PEACHES. WC = waist circumference. MVPA = moderate and vigorous physical activity (>4000 accelerometer counts per minute). Sedentary time (<100 accelerometer counts per minute).

Hierarchical linear regression B = unstandardised Beta value, β = standardised beta. Adjusted R^2^ for Step 1 = 0.83, p<0.001; Adjusted R^2^ for Step 2 = 0.84, Δ R^2 p =^ p = 0.237.

**Figure 2:**
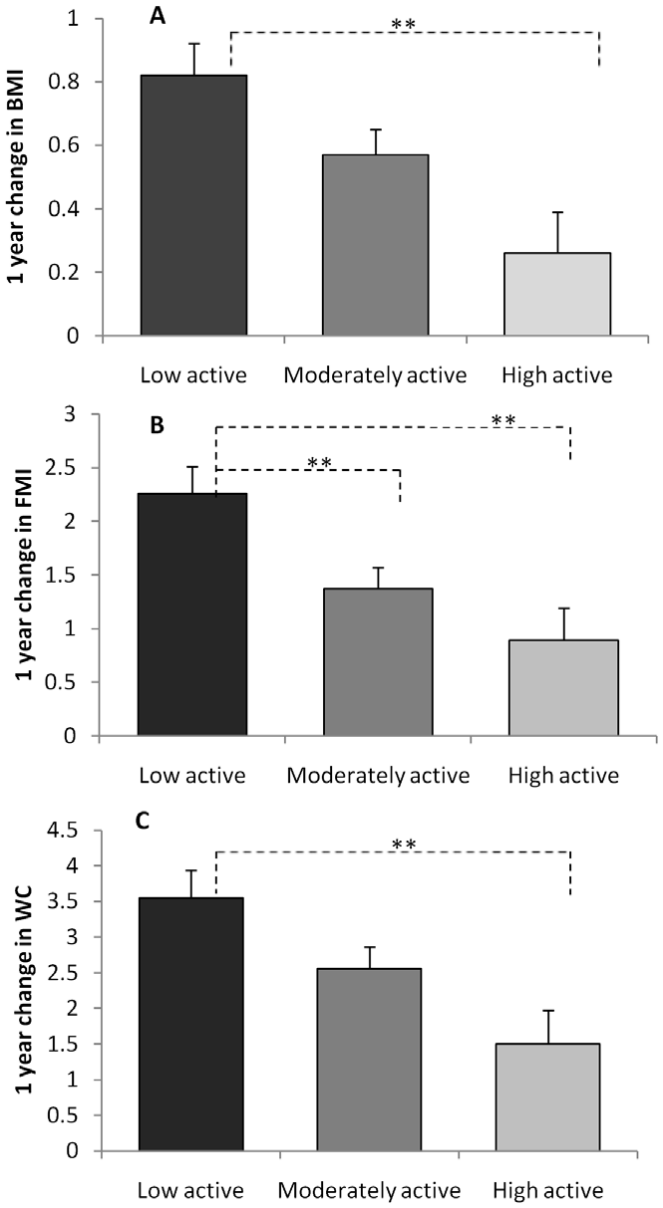
Adiposity by tertile of moderate and vigorous physical activity (MVPA; >4000 accelerometer counts per minute). Participants were 8–10 year old children participating in the PEACHES study. Data were mean change (follow-up minus baseline scores) adjusting for gender, SES, ethnicity, total physical activity and sedentary behaviour. Figure 2A displays 1 year change in body mass index (BMI; weight in kg/height in m^2^; n = 280 children). 2B 1 year change in fat mass index (FMI; fat mass/height in m^2^;n = 279 children) and 2C 1 year change in waist circumference (WC; n = 280 children). All linear trends were significant at p<0.01. Between group differences are highlighted; ** = p<0.01.

One year change in BMI, FMI and WC by tertiles of MVPA, adjusted for gender, SES ethnicity, total PA, and sedentary time are presented in **Figure 2**. The linear trends for BMI, FMI and WC were all significant (p's for trend all <0.01). Children in the highest tertile for MVPA (‘higher active’) had a significantly lower increase in BMI and WC than those in the lowest tertile (‘low active’) (BMI p = 0.008; FMI p = 0.008). Children in the middle tertile (‘moderately active) and higher active groups had significantly lower increases in FMI than those in low active group (p's<0.01).

## Discussion

The results of this study suggest that more time spent in MVPA was associated with lower adiposity one year later, independent of total physical activity or sedentary time. This finding gives an impetus to developing effective strategies for specifically promoting MVPA, as well as an overall active lifestyle, in children.

The finding that MVPA was more predictive of weight gain than total activity or sedentary time concurs with results from a well designed study involving adolescents, where stronger associations with follow-up fat mass were observed for activities with >3600 accelerometer counts per minute [12]. Recently, data from 405 children in the Gateshead Millenium project demonstrated that physical activity showed the steepest decline over time in children with higher BMI [30]. Both these UK-based studies involved predominantly white children. A strength of the PEACHES study is that our central London location allowed us to recruit an ethnically diverse sample. Significant cross-sectional associations between MVPA and body fat that were independent of total PA and sedentary behaviour have also been reported in 9–10 year olds [11], [31]. Demonstrating that these results hold longitudinally strengthens the case for a causal association.

It is not entirely clear why MVPA, but not total PA, was associated with lower gains in adiposity in our study. Levels of MVPA may be, to some extent, reflective of generally healthier lifestyle. However, there are biologically plausible mechanisms through which higher levels of physical activity could positively impact on body composition. Bouts of MVPA can elevate post-exercise resting energy expenditure for sustained periods [32]. Physical activity can also result in enhanced fat oxidation [32], [33] and improves fat distribution in children by reducing abdominal adiposity [34]. It is likely that vigorous physical activities would have a stronger effect; for example, one study involving 421 US adolescents demonstrated that activities >6 METS were more strongly negatively associated with fatness than moderate (4–6 METS) [9]. This concurs with another study using physical activity recall in which VPA was negatively associated with percentage body fat [35]. VPA levels in youth are also more predictive than total activity of adult physical activity levels [36]. It should be noted that, had it been possible, we would have examined MPA and VPA separately. However, in our sample of inner-city children the time spent in VPA was negligible. As in our study, the majority of other paediatric studies using objective measures also combine MPA and VPA, since habitual VPA in childhood is generally very low. Therefore, a focus on promoting activities that are at least moderate in intensity is an important target in children.

The association between MVPA and WC gain in our study was of borderline statistical significance (p = 0.06), but the effect size was similar to those for BMI and FMI. Furthermore, there was a significant WC trend across tertiles of MVPA, with children in the lower MVPA tertile demonstrating a greater increase in WC than those in the highest. One other longitudinal study in children failed to find an association between PA and WC in longitudinal analyses, [13] but the cut-point used to define MVPA (<2000 counts per minute) was lower than most studies that found significant associations, which may explain the discrepancy. It has also been suggested that the association between PA and central adiposity is moderated by fitness level [37]; a variable that was not measured in our study but could help explain variability in results across studies. Future research should assess fitness level as well as activity where possible.

The amount of the variance in BMI and FMI gain explained by MVPA was small in this study (in the region of 1%), but the time spent in MVPA was also extremely low (a mean of 12 minutes a day) and follow-up period relatively short. With a larger sample and a higher proportion of active children, the observed effect may be larger. It is also possible that associations between PA and weight status differ by age [38], and future research should follow children over longer periods and include important transitions such as from childhood to adolescence.

As in all longitudinal studies of adiposity [39], [40], weight status at baseline was the strongest predictor of weight status at follow-up; explaining more than 75% of the variance in this study. The strong tracking of adiposity over childhood is well-documented, but it may vary developmentally. In a study that followed children from 3 to 6 years, a model including baseline BMI and PA predicted only 65% of the variance in follow-up BMI [41], compared with 93% in our study. If the dominance of baseline weight is an indicator of the strength of tracking, it is possible that early interventions have a better chance of success.

Only 1% of the children in our sample met even the minimum guidelines of at least 60 minutes per day of MVPA [29]. While this seems extremely low, recent evidence using accelerometers in UK and US adults suggest that 95% of the population are not meeting the minimum adult activity guidelines of at least 30 minutes of MVPA per day [42], [43]. Objective evidence using accelerometers and doubly-labelled water in a large sample of UK preschool children suggests that even 3–5 year old children are sedentary for more than 80% of their waking hours [44]. Our results are even more concerning than another recent UK study of 9–10 year olds that found (albeit using a lower cut point (>2000 counts per minute) to define MVPA) that 30% of children not meeting the guidelines [45]. The urban context and ethnic diversity of our sample may contribute to these disturbing findings, but if the already low levels of activity decline further in adolescence, there are serious concerns for the health of the next generation of young adults.

The strengths of our study included an objective measure of PA, an understudied group, the use of three weight status indicators as opposed to BMI alone, and a longitudinal design. There were also a number of limitations. Fat mass index was measured with bioelectrical impedance which has relatively poor accuracy but the ‘gold standard’ four compartment model [46] is not feasible outside the clinical research context. The sample size was modest and levels of activity were very low. In a larger sample, a stronger relationship between total activity and weight status may have been observed [12], [31]. Finally, physical activity was also only measured at baseline, although it tends to be fairly stable from age 6 to 10 years [47].

### Conclusions

The results of this study make a case for promoting moderate and vigorous physical activity in childhood to protect against weight gain. They also argue for a better understanding of the relationship between activity levels and adiposity.
